# Supplementary material for: Direct time-resolved observation of surface-bound carbon dioxide radical anions on metallic nanocatalysts
Source: Nat Commun. 2023 Nov 6;14:7116. doi: 10.1038/s41467-023-42936-6 (PMC10628153; doi:10.1038/s41467-023-42936-6)
Supplement: Supplementary file 1 — Supplementary information [file 41467_2023_42936_MOESM1_ESM.pdf]

Supplementary information

Direct time-resolved observation of surface-bound carbon dioxide radical anions on metallic nanocatalyst

Zhiwen Jiang[1,2], Carine Clavaguéra[2], Changjiang Hu[3], Sergey A. Denisov[1], Shuning Shen[3], Feng Hu[3], Jun Ma[1]*, and Mehran Mostafavi[2]*

[1] *School of Nuclear Science and Technology, University of Science and Technology of China, Hefei, Anhui 230026, China.*

[2] *Université Paris-Saclay, CNRS, Institute de Chimie Physique, UMR8000, 91405, Orsay, France.*

[3] *Department of Materials Science and Technology, Nanjing University of Aeronautics and Astronautics, Nanjing, 211106, P. R. China.*

E-mail: majun0502@ustc.edu.cn; mehran.mostafavi@universite-paris-saclay.fr

Supplementary Table and Figures:

Supplementary Table 1: Structural and electronic properties of $(CO_2^{\bullet-})^{ad}_{Au}$ and $(CO_2^{\bullet-})^{ad}_{Cu}$, and comparison with $CO_2^{\bullet-}$ ADCH for atomic dipole corrected Hirshfeld atomic charges.

	$(CO_2^{\bullet-})^{ad}_{Au}$	$(CO_2^{\bullet-})^{ad}_{Cu}$	$CO_2^{\bullet-}$
**C-O (Å)**	1.231<br>1.230	1.305<br>1.248	1.233
**COC (deg)**	138	124	134
**M-C (Å)**	2.172	2.011	
**M-O (Å)**		2.080	
**$CO_2^{\bullet-}$ ADCH charge (e)**	-0.5	-0.6	-1.0
**M---C Mayer bond order**	0.45	0.85	
**M---O Mayer bond order**	-	0.54	
**M-C ELF basin**	1.2 e	2.2 e + 2.5 e	

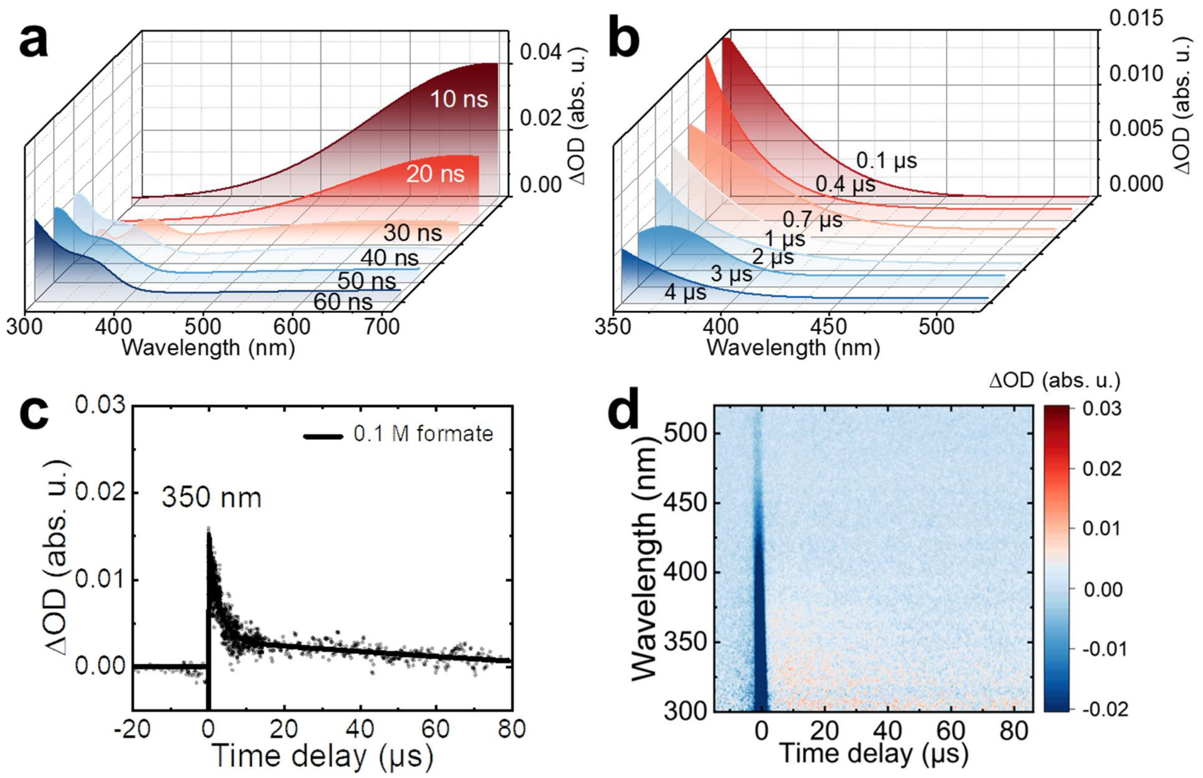

Supplementary Fig. 1 | Time-resolved absorption of $CO_2^{\bullet-}$ radical in free nanocatalyst solution containing 0.1 M formate. a-b, 3D stereographs of fitted transient absorption spectra within 60 ns (**a**) and 4 μs (**b**). **c,** Transient kinetics at 350 nm within 80 μs. **d,** Transient absorption matrix, showing the evolution of the absorbance at every wavelength versus time after one electron pulse. Source data are provided as a Source Data file.

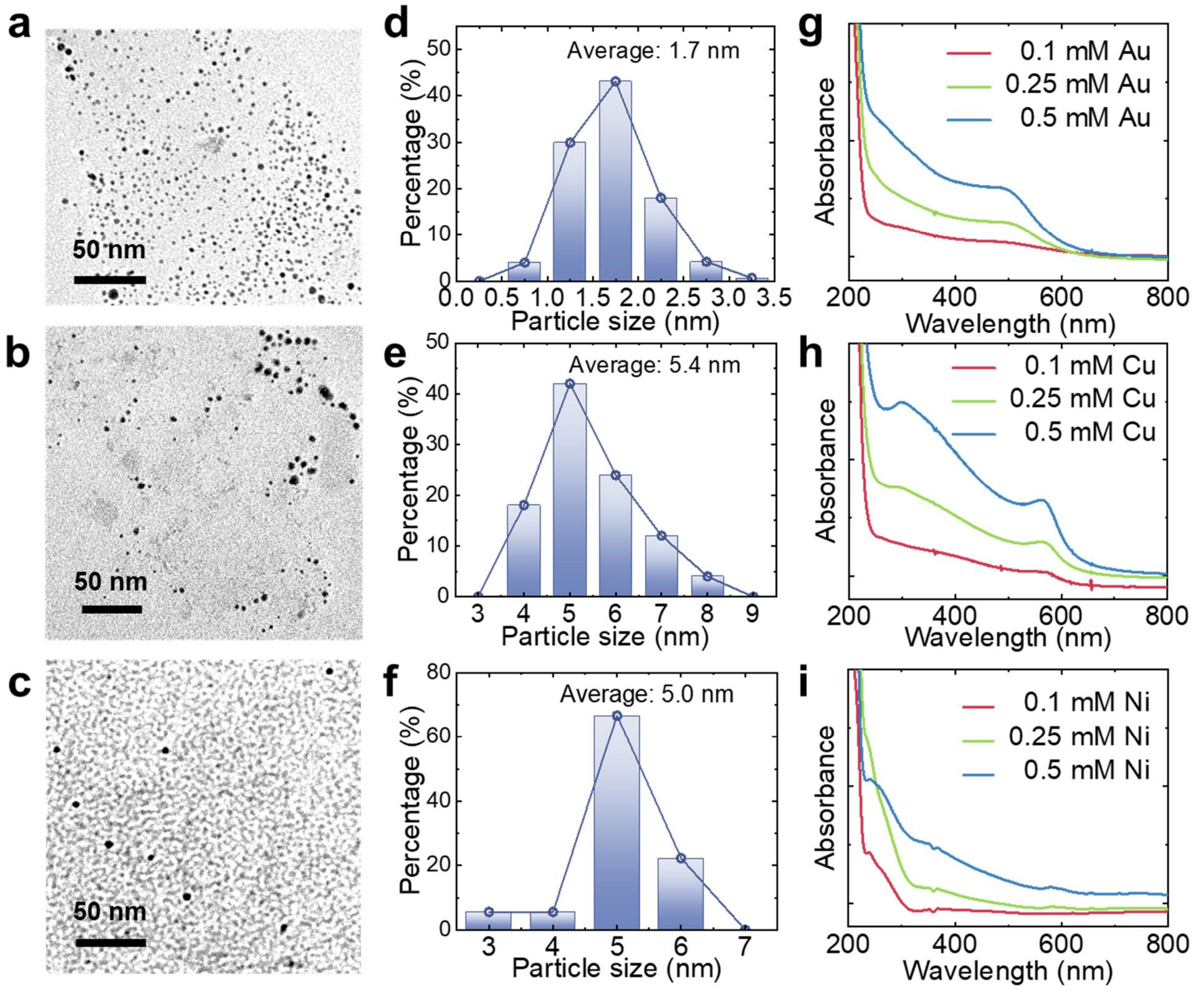

Supplementary Fig. 2 │ Morphology and size distribution of Au, Cu, and Ni nanoparticles. **a-c,** TEM image of Au (**a**), Cu (**b**), and Ni (**c**) nanoparticles. **d-f,** Size distribution derived from TEM images of Au (**d**), Cu (**e**), and Ni (**f**) nanoparticles. **g-i,** UV-vis spectra of Au (**g**), Cu (**h**), and Ni (**i**) solutions. Source data are provided as a Source Data file.

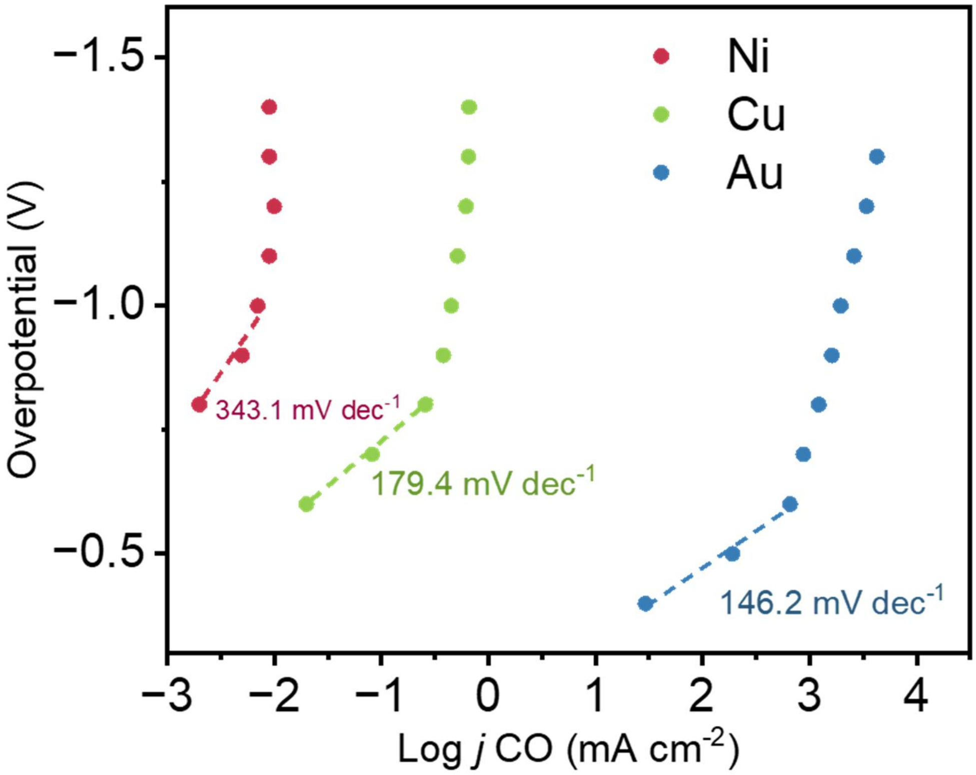

Supplementary Fig. 3 │Tafel plots for CO production catalyzed by Cu, Ni, and Au. Source data are provided as a Source Data file.

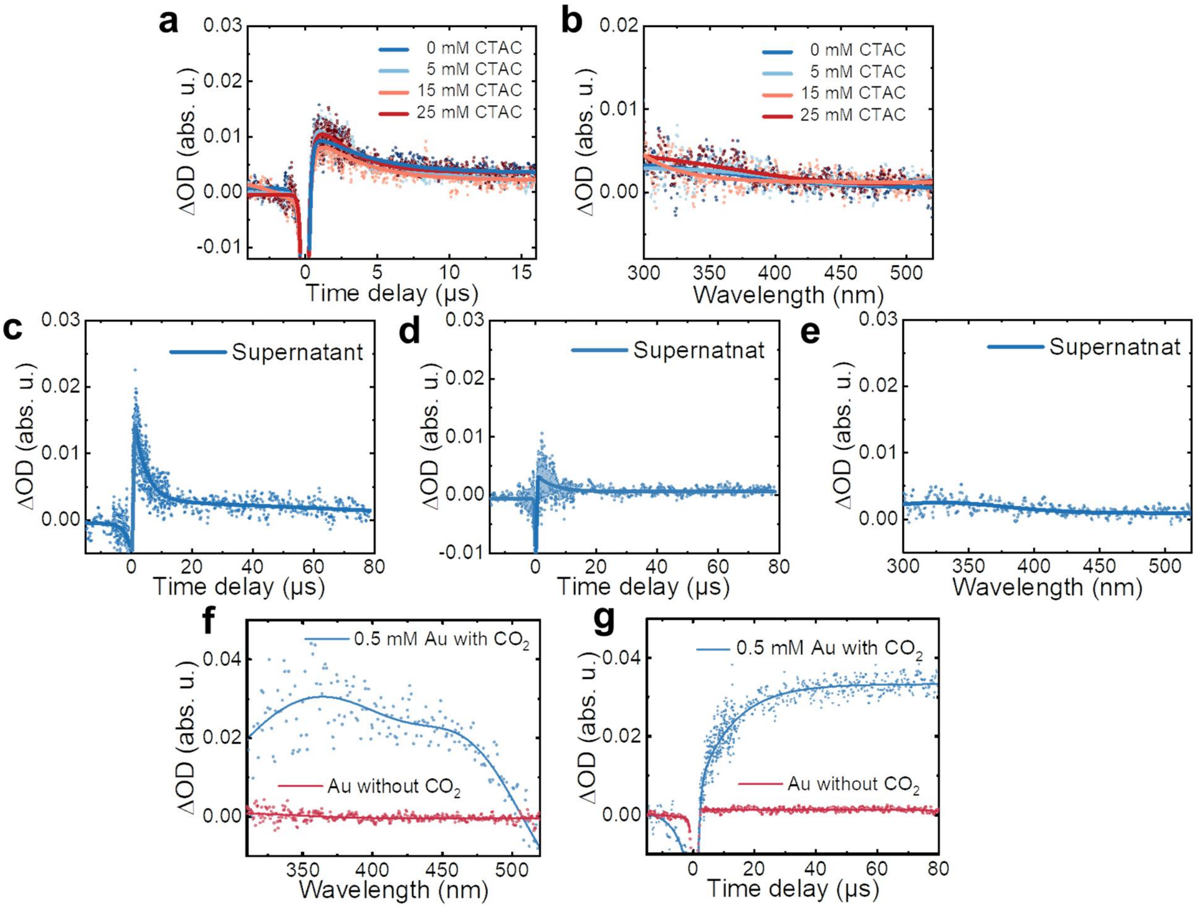

Supplementary Fig. 4 │ Transient kinetics and absorption spectra of $CO_2^{\bullet-}$ radical in reference solutions. a-b, Transient kinetics at 350 nm within 20 μs (**a**) and transient absorption spectra at 15 μs (**b**) in different concentration CTAC solutions. **c-e,** Transient kinetics at 350 nm (**c**) and 450 nm (**d**) within 80 μs and transient absorption spectrum at 80 μs (**e**) in the Au supernatant. **f-g,** Transient kinetics at 350 nm within 80 μs (**f**) and transient absorption spectrum at 80 μs in 0.5 mM Ar-saturated or CO_2-saturated Au solution. Source data are provided as a Source Data file.

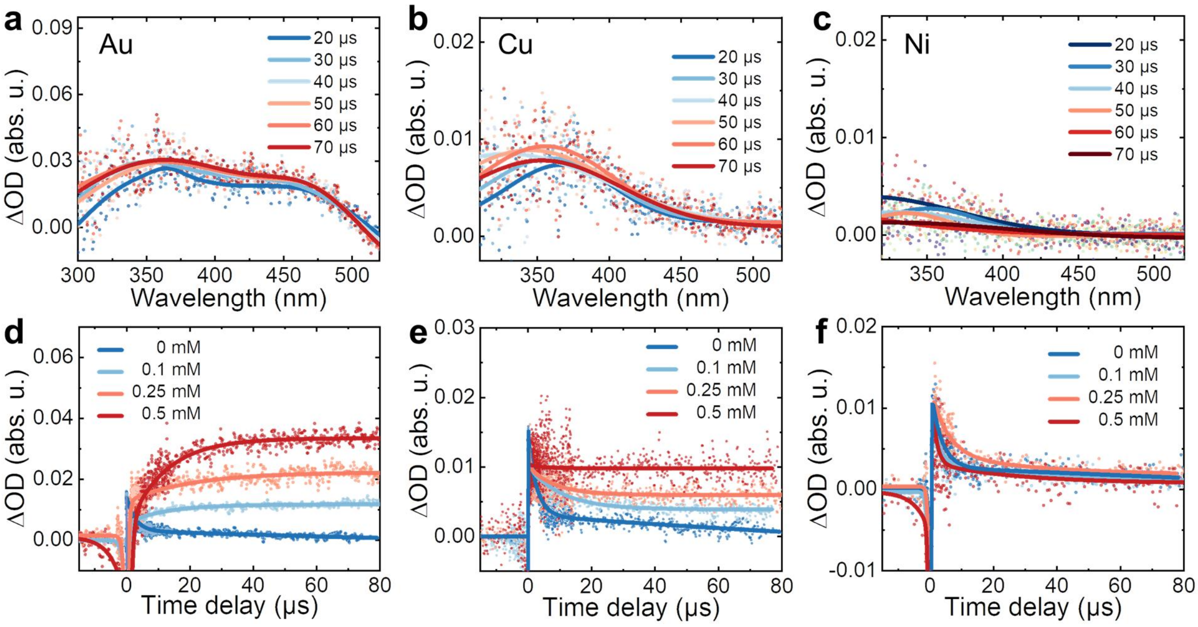

Supplementary Fig. 5 │ Transient kinetics and absorption spectra of $CO_2^{\bullet-}$ radical in Au, Cu, and Ni solutions. a-c, Transient absorption spectrum within 80 μs in 0.5 mM of Au (**a**), Cu (**b**), and Ni (**c**). **d-f.** Transient kinetics at 350 nm within 80 μs in the presence of different concentrations of Au (**d**), Cu (**e**), and Ni (**f**). Source data are provided as a Source Data file.

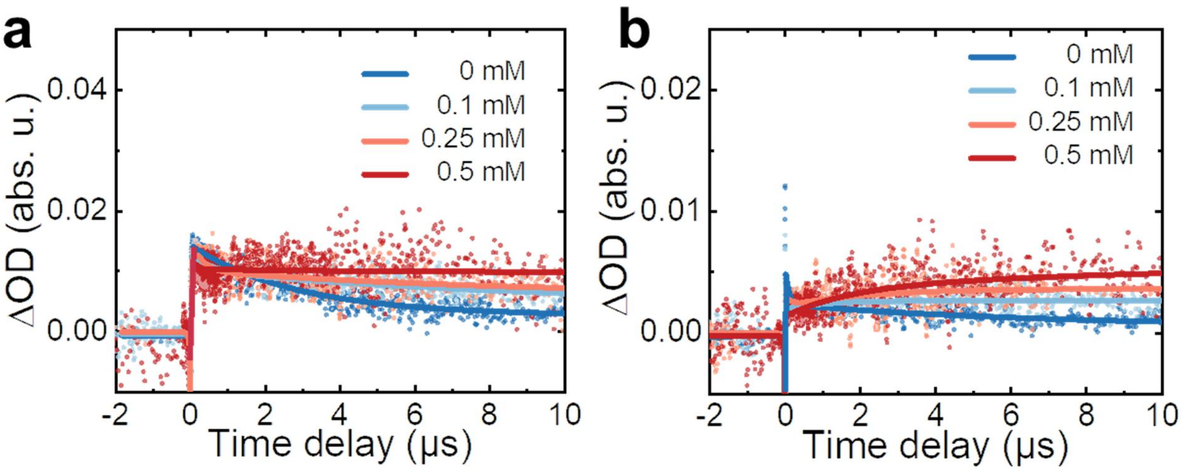

Supplementary Fig. 6 │ Transient kinetics of $CO_2^{\bullet-}$ radical stabilization process with different concentration Cu solution. a, Transient kinetics at 350 nm within 10 μs. **b,** Transient kinetics at 420 nm within 10 μs. Source data are provided as a Source Data file.

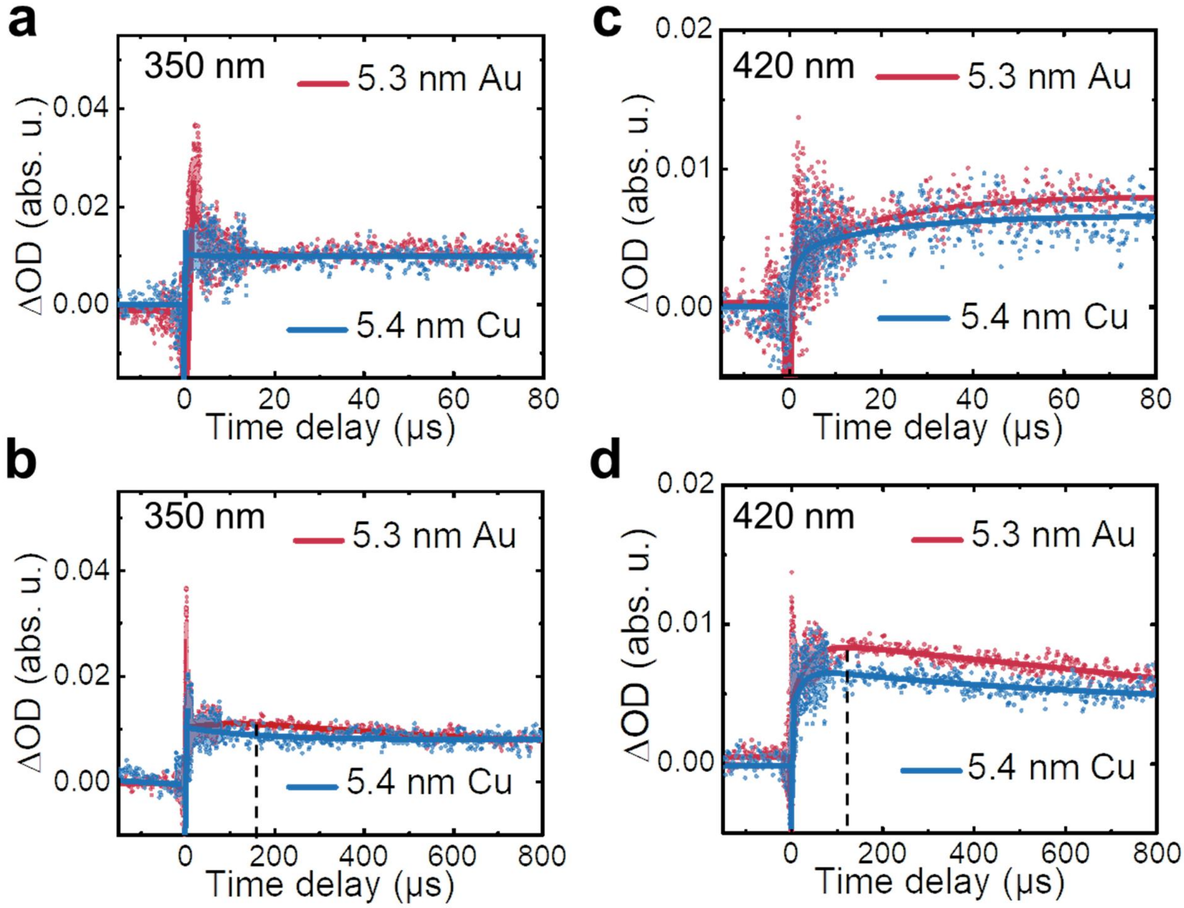

Supplementary Fig. 7 │ a-b. Transient kinetics at 350 nm with 5.3 nm Au and 5.4 nm Cu within 80 μs (**a**) and 800 μs (**b**) in 0.5 mM Metal NP solution. **c-d.** Transient kinetics at 420 nm with 5.3 nm Au and 5.4 nm Cu within 80 μs (**c**) and 800 μs (**d**) in 0.5 mM Metal NP solution. Source data are provided as a Source Data file.

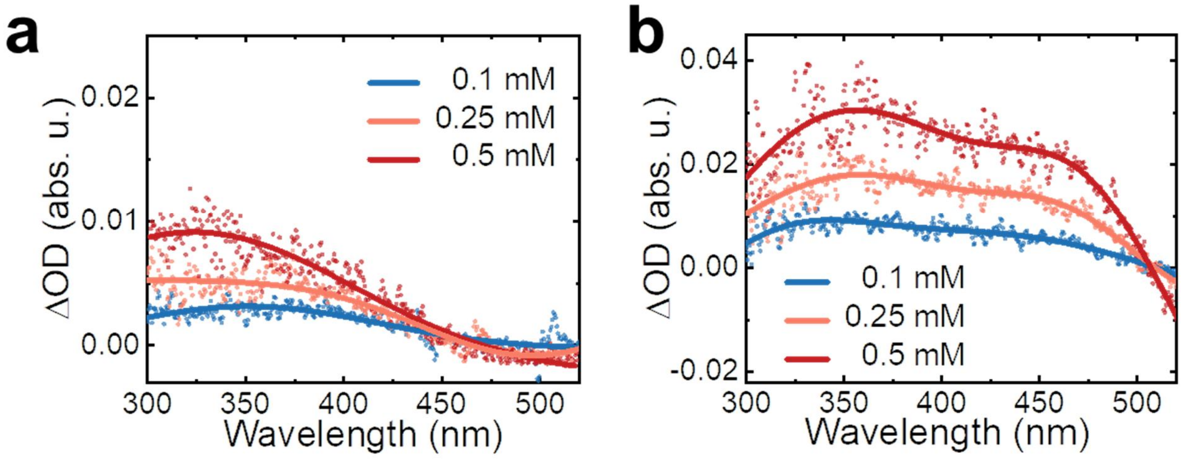

Supplementary Fig. 8 │ Transient absorption spectra of $(CO_2^{\bullet-})_{NP}^{ad}$ radicals at 750 µs in the presence of different concentration of Cu (a) and Au (b) NPs. Source data are provided as a Source Data file.

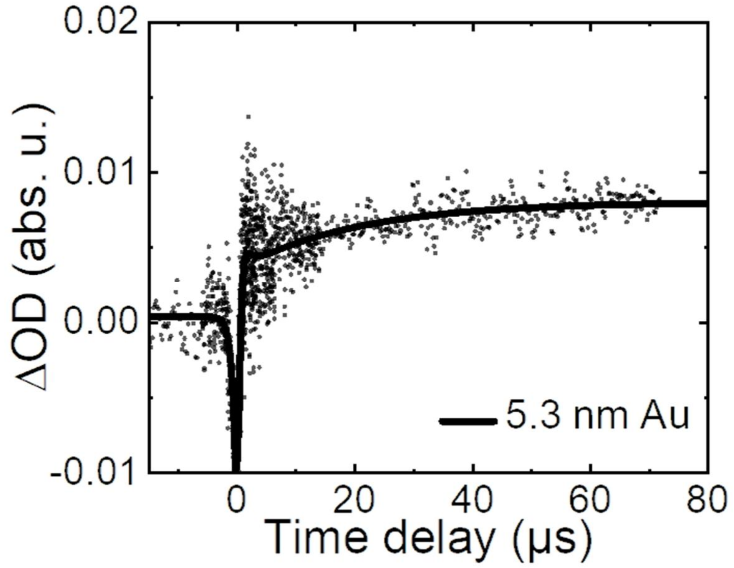

Supplementary Fig. 9 │ Transient kinetics of $(CO_2^{\bullet-})_{Au}^{ad}$ radicals at 450 nm in the presence of 0.5 mM 5.3 nm Au. Source data are provided as a Source Data file.

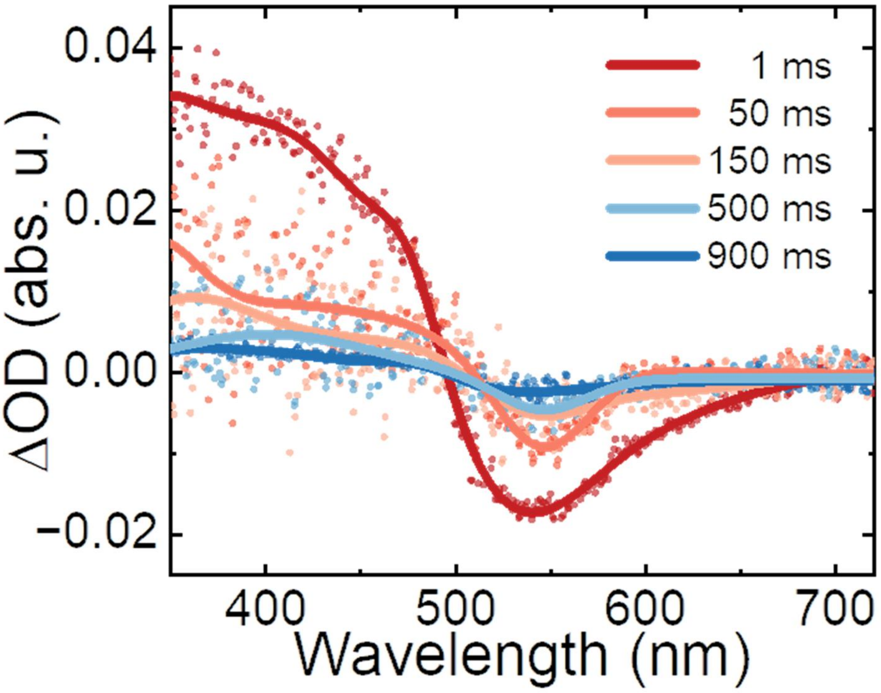

Supplementary Fig. 10 | Second timescale absorption profiles. Transient absorption spectra of $(CO_2^{\bullet -})^{ad}_{Au}$ radicals at 1 ms, 50 ms, 150 ms, 500 ms, and 900 ms in the presence of 0.5 mM 1.7 nm Au. Source data are provided as a Source Data file.

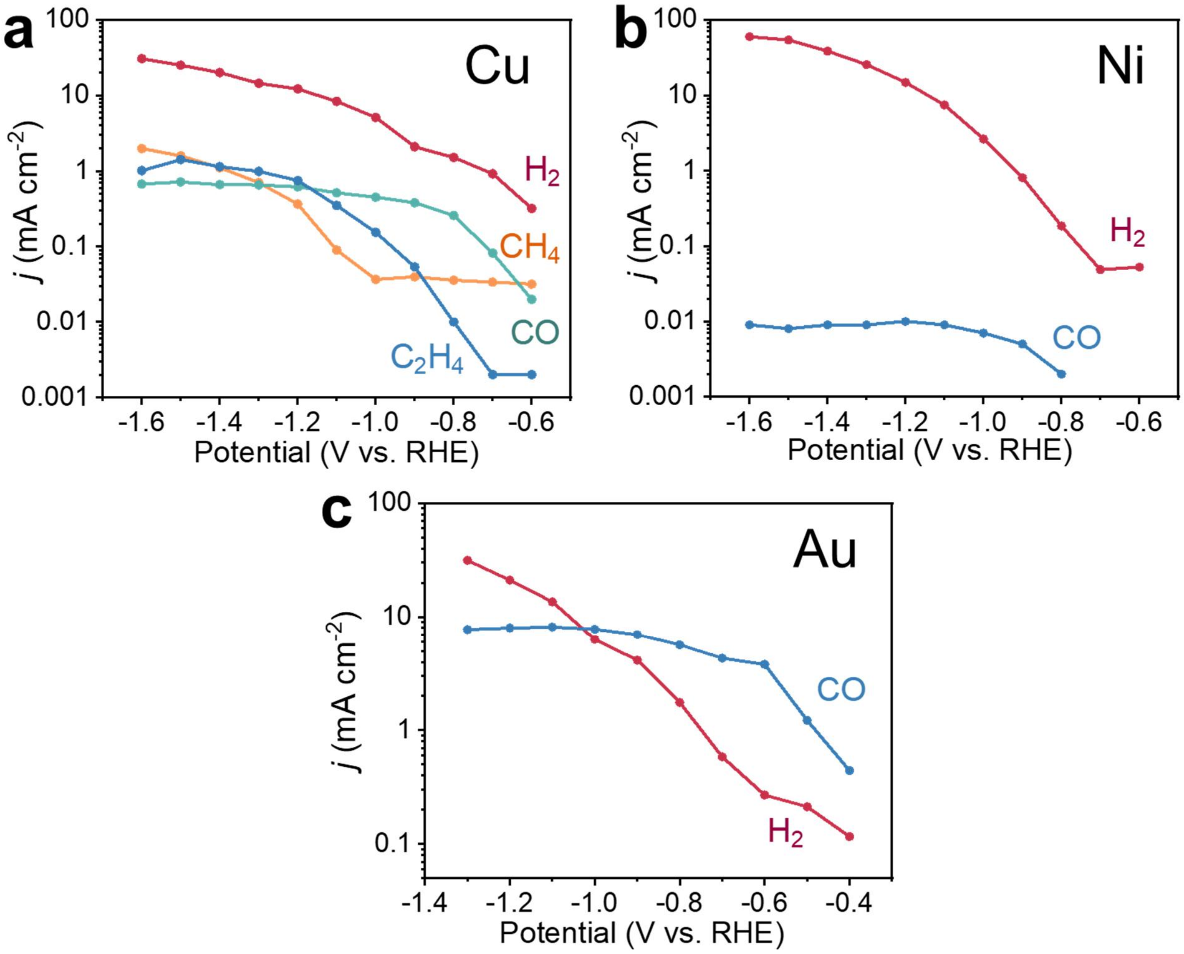

Supplementary Fig. 11 │ Partial current density for different products by Cu(a), Ni (b), and Au(c). Source data are provided as a Source Data file.

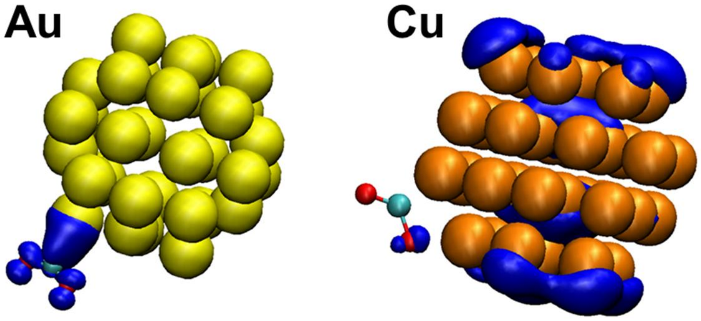

Supplementary Fig. 12 │ Total spin density of the ground state for of $(CO_2^{\bullet-})^{ad}_{Au}$ (left) and $(CO_2^{\bullet-})^{ad}_{Cu}$ (right). Source data are provided as a Source Data file.

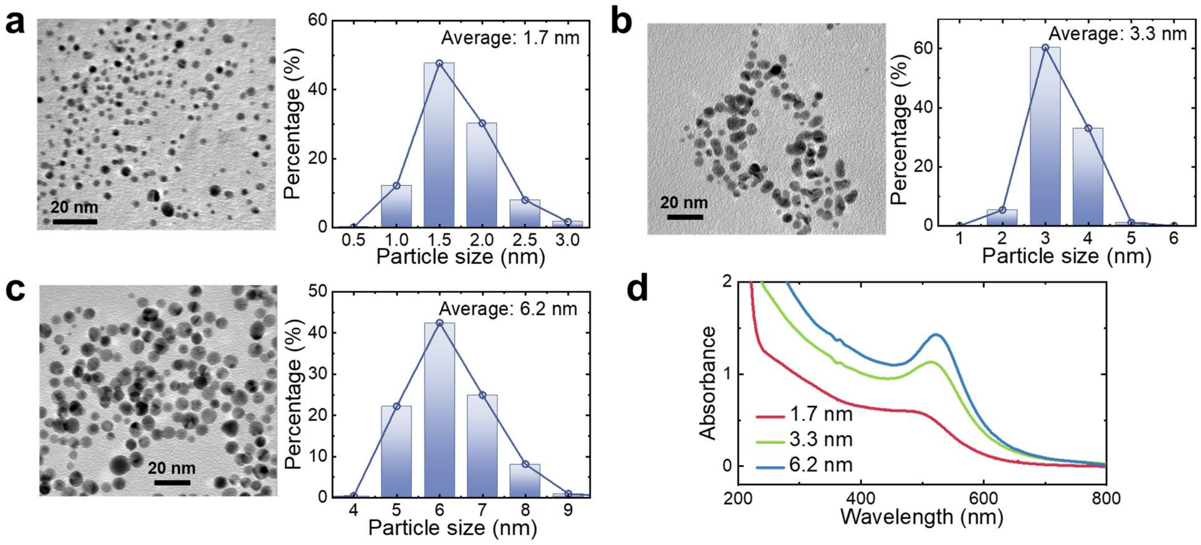

Supplementary Fig. 13 │ Morphology and size distribution of size-controlled Au nanoparticles. a-c, TEM image and corresponding size distribution of Au nanoparticles with the size of 1.7 nm (**a**), 3.3 nm (**b**), 6.2 nm (**c**). **d,** UV-vis spectra of Au dispersions. Source data are provided as a Source Data file.

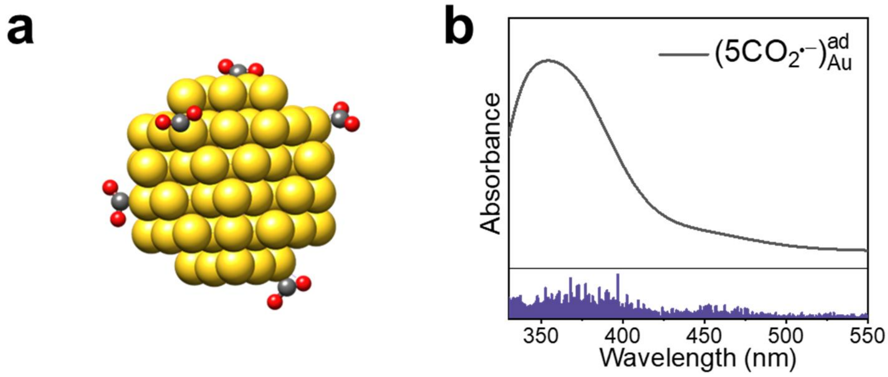

Supplementary Fig. 14 │ The simulation of 5 $CO_2^{\bullet -}$ radical stabilized on a 92-atom Au NP. **a**, DFT optimized structure. **b**, sTDA electronic spectrum of $(5CO_2^{\bullet -})^{ad}_{Au}$ (a shift of +80 nm has been applied to correct the sTDA approximation). Source data are provided as a Source Data file.

.

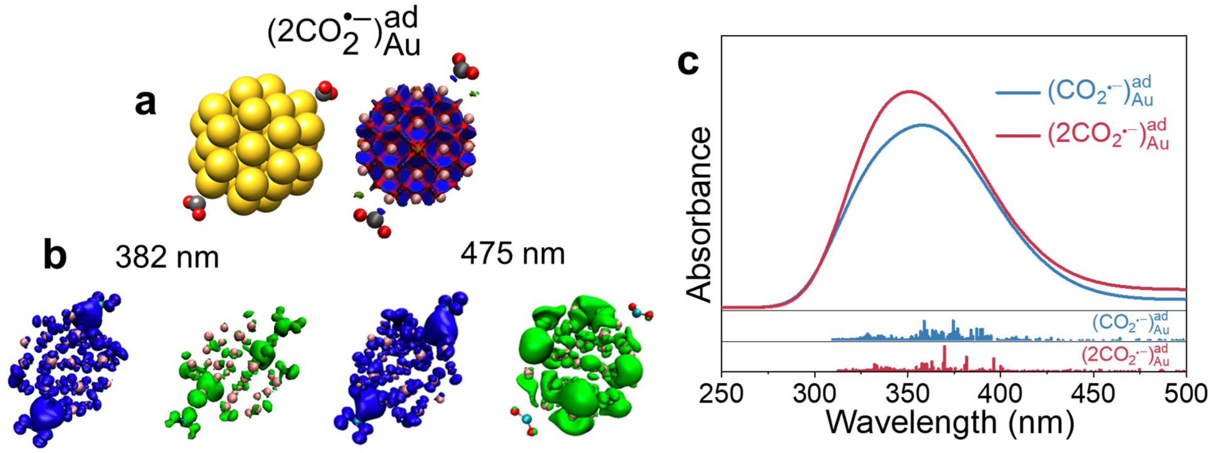

Supplementary Fig. 15 │ The simulation of 2$CO_2^{\bullet-}$ radical stabilized on Au. a-b, DFT optimized structures, DORI plots (repulsive forces in red and covalent interaction in blue) (**a**), and electronic transition analysis (hole in blue and electron in green) (**b**) of $(2CO_2^{\bullet-})^{ad}_{Au}$. **c**, TD-DFT electronic spectra of $(CO_2^{\bullet-})^{ad}_{Au}$ and $(2CO_2^{\bullet-})^{ad}_{Au}$. Source data are provided as a Source Data file.

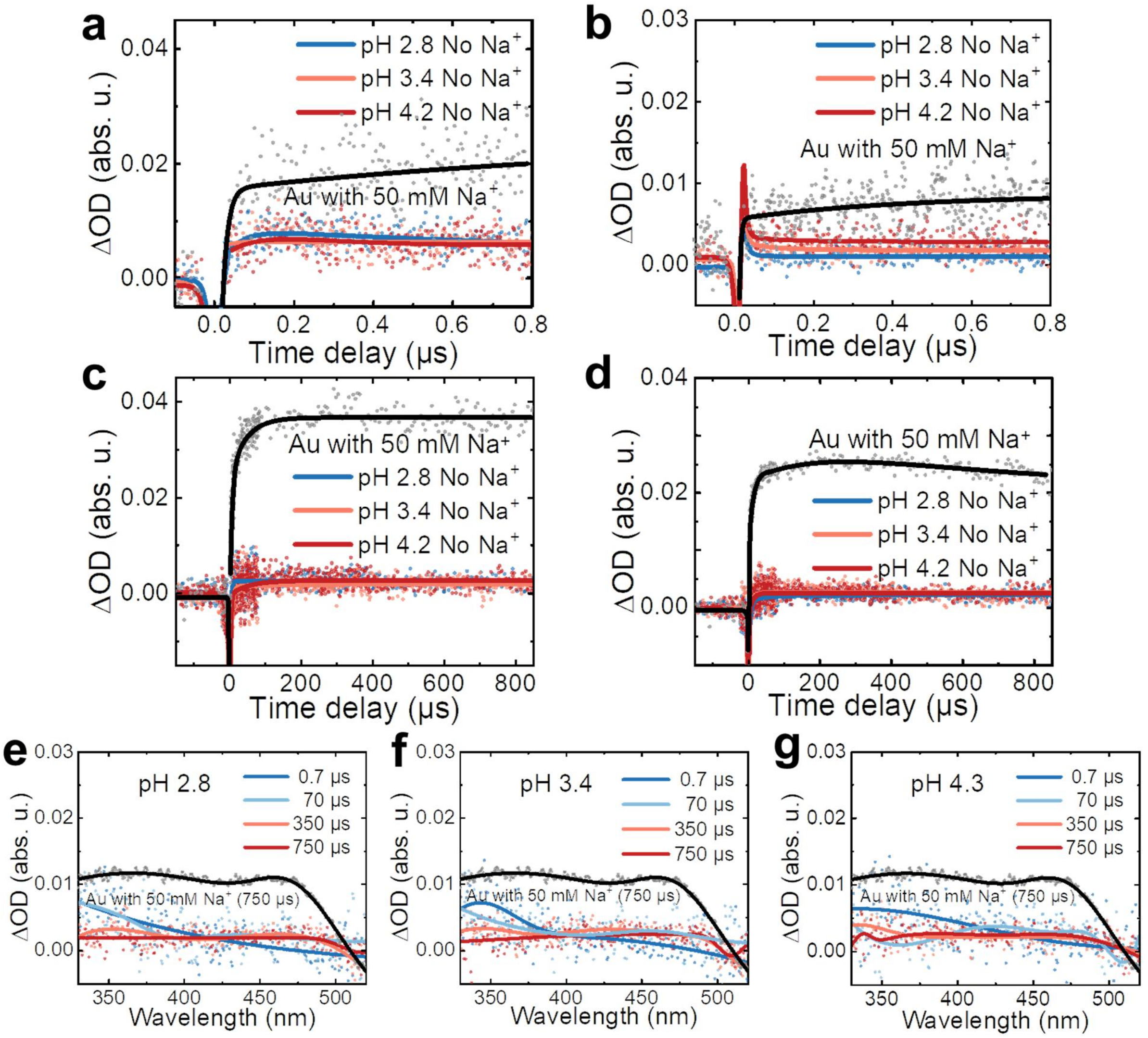

Supplementary Fig. 16 │ No metal cation on $CO_2^{\bullet-}$ radical stabilization process in the presence of 0.5 mM Au under different pH conditions within 750 µs. a-b, Transient kinetics within 800 ns at 350 nm (**a**) and 420 nm (**b**). **c-d**, Transient kinetics within 800 µs at 350 nm (**c**) and 420 nm (**d**). **e-g**, Transient absorption spectra at different times under pH 2.8 (**e**), pH 3.4 (**e**), and pH 4.2 (**g**). Source data are provided as a Source Data file.

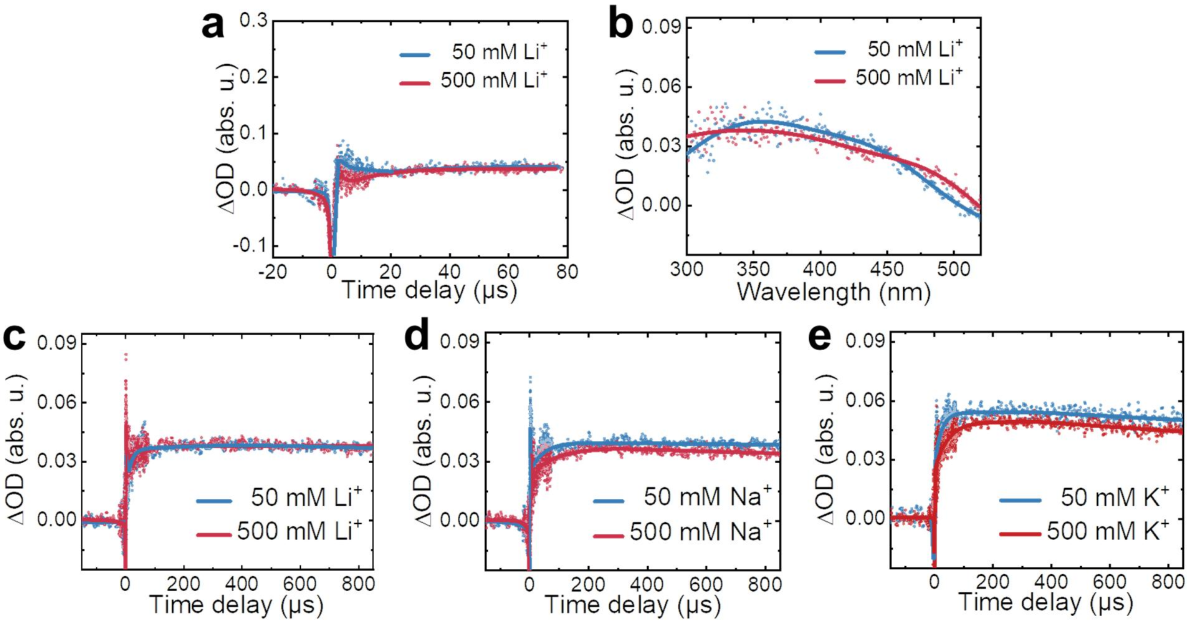

Supplementary Fig. 17 │ Cation effect on $CO_2^{\bullet-}$ radical stabilization process within 850 μs in 0.5 mM Au solution. a-b, Transient kinetics at 350 nm (**a**) and absorption spectra at 75 μs (**b**) of $*CO_2^{\bullet-}$ in different Li^+ concentration solutions (radiolytic preparation with lithium formate). **c-e,** Transient kinetics at 350 nm (**a**) of $*CO_2^{\bullet-}$ in different cation solutions within 850 μs. Source data are provided as a Source Data file.
